# Molecular modeling and characterization of *Vibrio cholerae *transcription regulator HlyU

**DOI:** 10.1186/1472-6807-6-24

**Published:** 2006-11-20

**Authors:** Rudra P Saha, Pinak Chakrabarti

**Affiliations:** 1 Department of Biochemistry, Bose Institute, P-1/12 CIT Scheme VIIM, Calcutta 700 054, India

## Abstract

**Background:**

The SmtB/ArsR family of prokaryotic metal-regulatory transcriptional repressors represses the expression of operons linked to stress-inducing concentrations of heavy metal ions, while derepression results from direct binding of metal ions by these 'metal-sensor' proteins. The HlyU protein from *Vibrio cholerae *is the positive regulator of haemolysin gene, it also plays important role in the regulation of expression of the virulence genes. Despite the understanding of biochemical properties, its structure and relationship to other protein families remain unknown.

**Results:**

We find that HlyU exhibits structural features common to the SmtB/ArsR family of transcriptional repressors. Analysis of the modeled structure of HlyU reveals that it does not have the key metal-sensing residues which are unique to the SmtB/ArsR family of repressors, yet the tertiary structure is very similar to the family members. HlyU is the only member that has a positive control on transcription, while all the other members in the family are repressors. An evolutionary analysis with other SmtB/ArsR family members suggests that during evolution HlyU probably occurred by gene duplication and mutational events that led to the emergence of this protein from ancestral transcriptional repressor by the loss of the metal-binding sites.

**Conclusion:**

The study indicates that the same protein family can contain both the positive regulator of transcription and repressors – the exact function being controlled by the absence or the presence of metal-binding sites.

## Background

The SmtB/ArsR family of metalloregulators is present in many bacteria and archaea, and its members respond to a variety of different metals. However, even in this well-studied regulator family, the determinants conferring metal specificity are only beginning to be understood [1]. Members of this family possess a highly conserved DNA recognition helix-turn-helix (HTH) domain and bind as homodimers to their operator/promoter (O/P) sequences, repressing the expression of operons associated with metal ion sequestration or efflux in both Gram-positive and Gram-negative bacteria, allowing these organisms to survive when challenged with toxic concentrations of heavy metal ions [2]. An evolutionary analysis, coupled with comparative structural and spectroscopic studies of six SmtB/ArsR family members, revealed that these proteins harbor one or both of the two structurally distinct metal coordination sites, denoted α3N or α5 [3,4], named for the location of the metal binding sites in the known or predicted secondary structural elements of individual family members. The members most closely related to a common ancestor, represented by the ArsR family, contain only α3N metal binding site, while the more divergent homologue CzrA possesses only α5 metal binding site. The α3N site is cysteine thiolate-rich, forming S_3 _or S_4 _complexes with large, thiophilic metals including Cd, Pb and Bi, as found in the cadmium sensor *Staphylococcus aureus *CadC [4-6], while site-directed mutagenesis and amino acid sequence comparisons suggested that the α5 site is composed of a combination of carboxylate and imidazole ligands, interacting preferentially with transition metal ions including Zn, Co and Ni [7-9]. Apart from α3N and α5 sites there are other metal sensory sites found in *Mycobacterium tuberculosis *H37Rv CmtR [10,11]. CmtR is a Pb(II)/Cd(II)-sensing SmtB/ArsR metalloregulatory repressor that lacks both typical α3N and α5 sites and possesses a novel metal-sensing site at the α4 or the DNA-recognition helix [11].

*Vibrio cholerae *expresses virulence factors that allow it to colonize the human intestine and cause the disease cholera. Regulation of virulence genes in *Vibrio cholerae *involves the ToxR, Fur, and HlyU regulatory systems [12]. The HlyU regulator controls the expression of HlyA [13], and a *hlyU *mutation attenuates *Vibrio cholerae *O17 in the infant mouse cholera model [14]. In addition to the well-studied role of the positive regulation of the transcription of HlyA, the possibility of HlyU controlling the expression of virulence determinants was suggested by the reduced colonizing ability of a *hlyU *mutant compared with that of a *hlyA *mutant. A recent study also showed that HlyU is one of the master regulators of *in vivo *virulence expression in *Vibrio vulnificus *[15]. Therefore, the HlyU protein itself and the genes under its control would serve as important targets in developing a new-paradigm therapy against *Vibrio cholerae*. Using the method of protein fold-recognition we found that *Vibrio cholerae *HlyU exhibits a domain common to the SmtB/ArsR family. The fact that all the members of SmtB/ArsR family are transcriptional repressors, in contrast to *V. cholerae *HlyU being a positive regulator of transcription, suggests that the knowledge of sequence-structure-function relationship in HlyU is desired. The elucidation of the three-dimensional (3D) structure could provide insight into molecular functions as well as evolutionary relationship between HlyU and the SmtB/ArsR family members. In this paper we elucidate the structure and mechanism of action of *V. cholerae *HlyU using computational methods.

## Results and Discussion

### Identification of the three-dimensional fold

To create a model of Vc-HlyU, we first performed a BLAST search for proteins with similar sequence and known 3D structure using the 108 residue long Vc-HlyU sequence (SWISS-PROT: P52695). Significant similarities were found with several ArsR family of transcription regulators (Table 1) suggesting that Vc-HlyU may belong to the same family. Additionally, a conserved domain search [16] of Vc-HlyU sequence also supported the above idea. A PROSITE analysis of Vc-HlyU sequence also indicated the presence of ArsR-type HTH domain signature and profile. In addition, a BLAST search against the structures in Protein Data Bank (PDB) also identified ArsR-like transcription regulators, though with lower *E*-values and ~35% sequence identity (Table 1). The proteins are CadC from *Staphylococcus aureus *pI258, CzrA from *Staphylococcus aureus*, and SmtB from *Synechococcus *sp., which could be considered as possible templates for modeling of Vc-HlyU using the threading approach, which allows to assess the compatibility of the target sequence with the available protein folds based not only on the sequence similarity but also on structural considerations [17,18]. The Vc-HlyU sequence was therefore submitted to the Genesilico protein fold-recognition metaserver. Fold-recognition servers SPARKS, FUGUE, and 3DPSSM reported SmtB from *Synechococcus *(PDB entry: 1smt) as the best template with highly significant score. INBGU scored CzrA from *Staphylococcus aureus *(PDB entry: 1r1u) as the best template, which was CadC from *Staphylococcus aureus *pI258 (PDB entry: 1u2w) according to mGENETHREADER. Fold-recognition alignments reported by these servers were compared, evaluated, and ranked by Pcons server, which assigned highly significant scores to *Synechococcus *PCC7942 SmtB (1^st^), *S. aureus *CzrA (2^nd^), and *S. aureus *pI258 CadC (3^rd^) as the potential modeling templates. The analysis by CATH and SCOP on the crystallographic structures of *Synechococcus *PCC7942 SmtB, *S. aureus *CzrA, and *S. aureus *pI258 CadC confirmed that they all belong to the 'Winged helix DNA-binding domain' superfamily, and 'ArsR-like transcriptional regulators' family. Their fold is well conserved in the entire superfamily, even if the sequence identity between the proteins in this family is low (~25–50%). On the basis of these results, we can conclude that Vc-HlyU has a fold similar to these proteins. Therefore, we used these experimental three-dimensional structures as templates to predict the 3D structure of Vc-HlyU by using comparative modeling strategy.

### Comparative modeling of the Vc-HlyU

**Figure 1:**
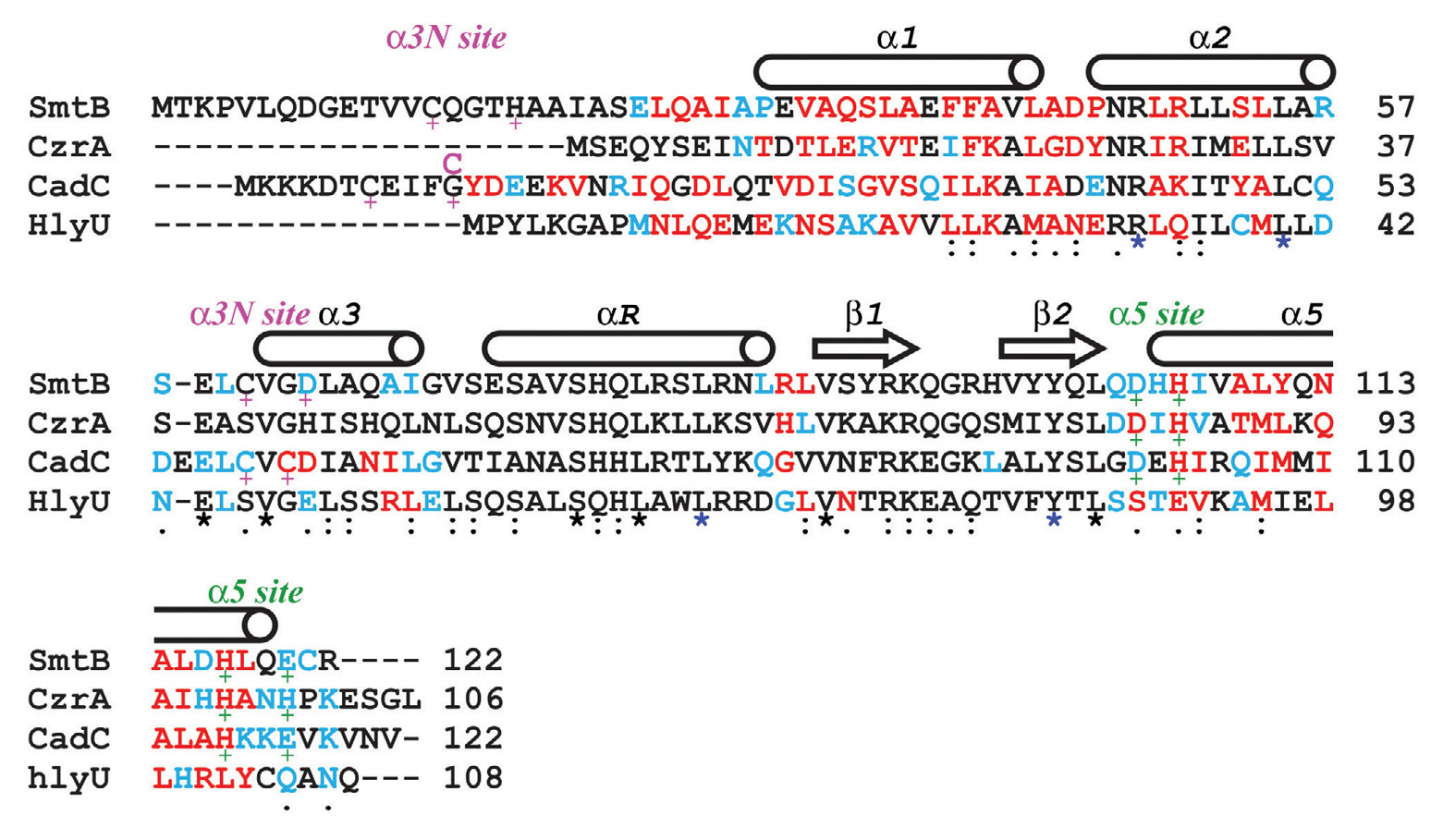
Alignment of the Vc-HlyU sequence with those of templates SmtB from *Synechococcus *PCC7942, CzrA from *Staphylococcus aureus*, and CadC from *Staphylococcus aureus *pI258. Secondary structural elements are shown (α-helices as cylinders and β-strands as arrows) and labeled (α4 is the DNA-recognition helix, αR). Metal binding sites α3N and α5 are marked with plus (+) sign in magenta and green color, respectively. Residues which formed the homodimeric interface are shown in colors, core-residues and rim-residues [37] are shown in red and sky-blue, respectively. Conserved residues are denoted by asterix (*), while residues conserved among all the SmtB/ArsR family members are colored in blue asterix (*). The wild-type CadC has a Cys in position 11, which was Gly in the recombinant protein.

The availability of experimental 3D templates allowed us to create a 3D model of Vc-HlyU by using the comparative modeling strategy, taking into account the difficulties encountered with low sequence identity (between 20 and 40%) – a borderline case that has to be treated carefully [19-22]. Nevertheless, when proteins used for alignment and modeling belong to the same family in which the structure is well conserved, functional information and overall structure similarity can overcome the problem of low-sequence identity and a good multiple sequence alignment can be obtained, suitable for applying the comparative modeling procedure [19]. Moreover, information such as the position of secondary structure elements can be used to verify the quality of the sequence alignment and to optimize the position of the gaps. Therefore, we performed a multiple sequence alignment, on Vc-HlyU and the proteins selected by fold-recognition methods. The positions of the experimentally observed secondary structure elements of templates and of the predicted secondary structure of Vc-HlyU were then superimposed onto the aligned sequences. We found good agreement between the predicted and experimental secondary structure positions. These results confirmed the good quality of the multiple alignment obtained, and after a few minor manual refinements to correct gaps, we used the final alignment (Figure 1) as the starting point to predict the 3D structure of Vc-HlyU.

To create the 3D model of Vc-HlyU, two sets of ten structural models were created using MODELLER in two distinct sessions, one set using SmtB from *Synechococcus *PCC7942 and CzrA from *Staphylococcus aureus *as templates, while the other set also additionally used CadC from *Staphylococcus aureus *pI258. We then performed a PROCHECK analysis on the stereochemical quality of the 20 models, selected the best model from each session, and used them as templates to generate a new set of ten models. The most reliable of them in terms of stereochemical quality (based on PROCHECK) was selected as the final model, which had 96.3% of residues in most favoured regions, 3.2% of residues in additional allowed regions and one residue in generously allowed regions with no residues in disallowed regions, a result expected for crystallographic models with at least 2.0 Å resolution and the R factor lower than 20% [23]. The side-chains of the models were optimized using SCWRL 3.0 program. A complete validation analysis on the final homodimeric model was also performed with ADIT Validation server at RCSB (Research Collabatory for Structural Bioinformatics) and confirmed its good quality. The model was also evaluated at the Eval123D server [24] using Eval23D [25], Verify3D [26], ProsaII [27], and Solvation Free Energy (SFE) of folding [28]. Results shown in Table 2 indicate that the quality of the model is as good as the crystallographically determined structures in the SmtB/ArsR family. Furthermore, the plot of Prosa values, representing the interaction energy of each residue with the rest of the protein, are negative along the whole sequence (see additional file 1), also indicating the overall reliability of the model.

### The three-dimensional structure of Vc-HlyU

**Figure 2:**
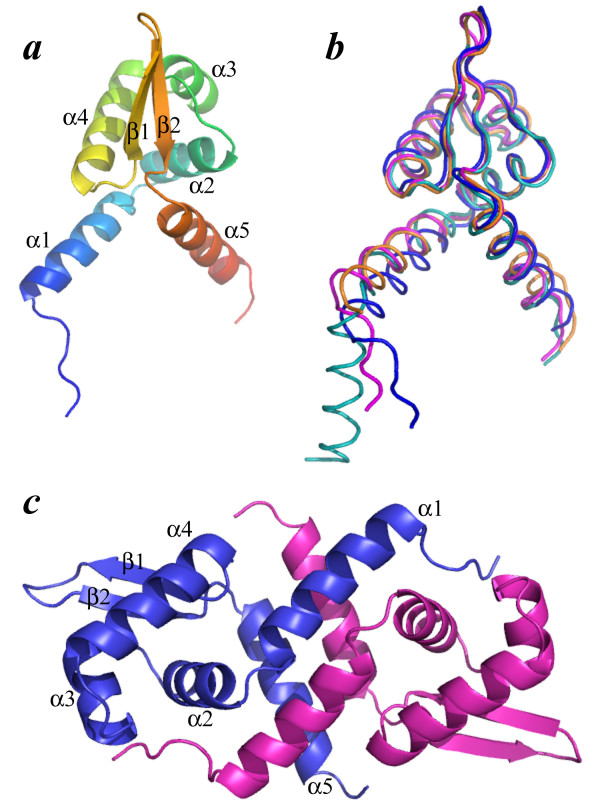
Three-dimensional structure of Vc-HlyU. (a) Cartoon diagram of Vc-HlyU monomer showing the α1-α2-α3-α4-β1-β2-α5 fold. (b) Superimposition of *Synechococcus *SmtB (blue color), *S. aureus *CzrA (orange), *S. aureus *CadC (teal), and Vc-HlyU (magenta) monomers. (c) Ribbon diagram of Vc-HlyU dimer showing the secondary structural units. Monomer A and B are in magenta and blue colors, respectively. All figures were created with PyMOL [73].

Each monomer of Vc-HlyU has a fold consisting of five α-helices and a pair of antiparallel β-strands in the topology α1-α2-α3-α4-β1-β2-α5 (Figure 2a). As expected the model is perfectly superimposable with the templates (Figure 2b), the root-mean-square deviations (RMSD) using Cα atoms being 2.04 Å, 0.32 Å and 0.85 Å with SmtB, CzrA and CadC, respectively (for comparison, the RMSD values between the template structures are SmtB-CzrA: 2.29 Å, SmtB-CadC: 3.29 Å and CzrA-CadC: 1.88 Å). Helices 3 (α3) and 4 (α4) constitute the helix-turn-helix motif in Vc-HlyU and the β-sheet is the wing as found in other winged-HTHs. Helix 4 (α4) is termed as the recognition helix (αR), like in other HTHs where it binds the DNA at the major groove. This helix-turn-helix domain (α3-turn-αR) has strong structural resemblance to other bacterial transcriptional regulators including CAP (catabolite activator protein) [29], and DtxR (the Fe(III)-regulated diphtheria toxin repressor) [30]. This DNA binding domain, particularly the sequence of the proposed DNA-recognition α-helix (αR), is also highly conserved throughout the SmtB/ArsR family and is one of the distinguishing characteristics that define membership. Most SmtB/ArsR-like metalloregulators form homodimers. The dimer interface is formed by helix 5 (α5) and an N-terminal part of the protein [31], as can also be seen in the model of Vc-HlyU (Figure 2c).

HlyU has two cysteine residues (C38 and C104, Figure 1), not linked by any disulfide bridge; C104 (in α5) is solvent exposed, while C38 (in α2) is partially buried, as was indicated by biochemical studies [32].

### Vc-HlyU does not have the key metal-binding residues

Comparative biochemical, spectroscopic, and theoretical studies on SmtB/ArsR family members, reveals that these proteins possess one or both of two structurally distinct metal-binding sites, denoted by α3N or α5, named according to the location of the metal sites in the known or predicted secondary structure of individual family members. Metal binding leads to derepression by inducing a conformational change leading to the release of the metalated repressor from the O/P sequence. In the case of CadC, binding of the metal brings the N-terminus of one subunit into position to sterically block the DNA binding site of the other subunit [33]. *Synechococcus *PCC7942 SmtB binds Zn^2+^, has both the α3N and α5 metal binding sites, but only the α5 site is functional; N-terminal residues C14, H18, and residues C61 and D64 (in the N-terminus of α3 helix) comprise the first metal binding site or α3N site; while the other Zn^2+^-binding site or the α5 site occurs at the dimer interface between the C-terminal α5 helices of two monomers, they are formed by two residues from each monomer, D104 and H106 from one monomer along with H117 and E120 from the other [31] (Figure 1). *S. aureus *pI258 CadC has a similar structural arrangement, α3N site consists of C7, C11, C58, and C60 residues, while α5 site consists of D101, H103, H114, and E117 residues. *S. aureus *CzrA does not have the α3N site, but the α5 site is present and consists of D84, H86, H97, and H100 residues in the same structural arrangement as in the other members of SmtB/ArsR family of proteins.

**Figure 3:**
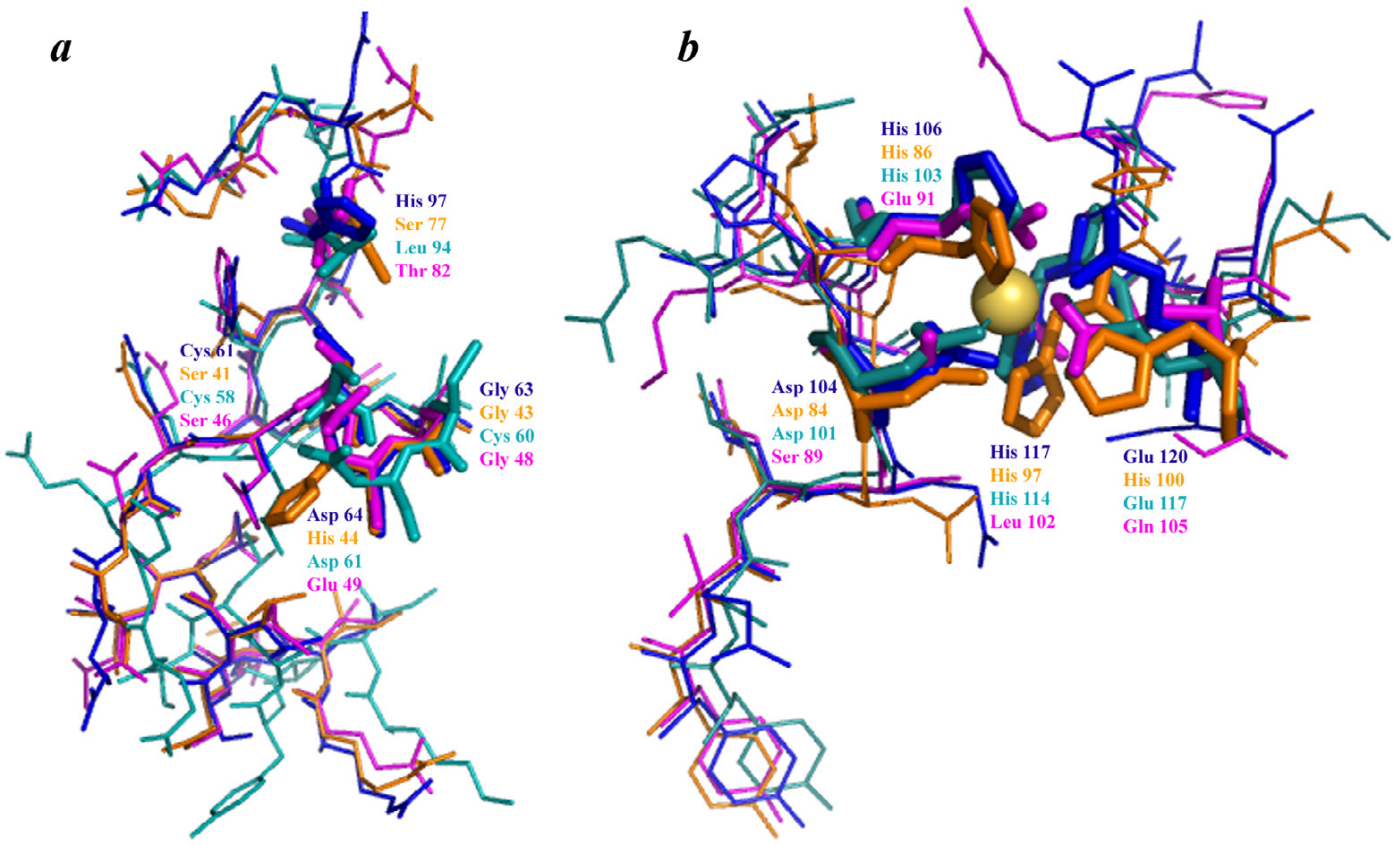
Superimposition of (a) α3N and (b) α5 metal binding sites of *Synechococcus *SmtB (blue color), *S. aureus *CzrA (orange), *S. aureus *CadC (teal), and Vc-HlyU (magenta).

A highly conserved ELCV(C/G)D motif termed as the 'metal binding box' was initially identified in members of the SmtB/ArsR family [2]. This motif was proposed to contain residues involved in metal coordination and, therefore, directly involved in metal ion sensing. SmtB and CadC have this motif, ^59^ELCVGD^64 ^and ^55^ELCVCD^60^, respectively in the α3 helix (Figure 1), as part of the projected α3-turn-αR DNA-binding motif [31,33]. This sequence is required for metal ion sensing by the direct binding of metal ions, suggested by the fact that the substitution of one or both cysteines with non-metal-liganding residues in the ^30^ELCVCD^35 ^motif inhibited the ability of arsenate salts to dissociate ArsR from the *ars *O/P [34]. Vc-HlyU has the the 'metal binding box' ^44^ELSVGE^49 ^(Figure 1) exactly in the same position as found in other SmtB/ArsR family members, but interestingly, the key metal sensor residue cysteine in this box is replaced by non-metal-ligand residue serine, indicating that Vc-HlyU may not bind metal at the α3N site. *S. aureus *CzrA, which does not bind metal at the α3N site, has similar motif ^39^EASVGH^44 ^as Vc-HlyU. Vc-HlyU also lacks the two metal binding residues present in the N-terminus of the protein as found in case of SmtB and CadC. This is similar to CzrA, which also does not have those metal-sensing residues. Therefore, the absence of N-terminal metal-binding residues in addition to the presence of non-metal binding residue serine in the 'metal binding box' suggests that Vc-HlyU does not bind any metal at the α3N site (Figure 3a).

The α5 metal site consists of four metal ligands derived exclusively from the two ends of α5 helix, forming a tetrahedral or distorted tetrahedral metal complex across the dimerization interface, as originally hypothesized from the crystallographic studies of SmtB [31]. Mutagenesis of H105 and H106, together, in SmtB had earlier been shown to inhibit Zn(II) sensing *in vivo*, suggesting that the metal site across the α5 helix may be more important for metal sensing by SmtB, in contrast to ArsR [35]. When we looked at the α5 metal-binding region in Vc-HlyU we found that again the key metal-binding residues are replaced by non-metal-binding residues, while keeping the overall structure similar to other Smtb/ArsR family members. The four conserved residues Asp, His, His, and Glu/His which formed the α5 metal binding site in SmtB, CadC, and CzrA (Figure 1) are replaced mostly by non-metal-binding residues Ser, Glu, Leu, and Gln, respectively, in Vc-HlyU, suggesting that Vc-HlyU also does not bind metal at the α5 site (Figure 3b). We also considered the possibility if other residues, such as E97, H100 and C104 around the α5 helix can be involved in metal binding. But these are positioned in a linear fashion at the hydrophilic side of the helix, opposite to the face involved in the dimeric interface. As such, even if these sites were to bind metal ions the dimeric interface will get disrupted.

*M. tuberculosis *CmtR is proposed to bind Pb(II) and Cd(II) via coordination by C57, C61, and C102 [11]. The C57 and C61 residues are in α4 DNA-recognition helix of CmtR while C102 is at the C-terminal end of the protein. Vc-HlyU does not have any cysteine residues at the α4 helix, but it has a cysteine (C104) residue at the C-terminal end. Therefore, Vc-HlyU also lacks the unique metal-sensory sites at the α4 helix as found in case of CmtR. Another protein *Streptomyces griceus *SrnR which showed homology to the transcription regulators of ArsR family represses the transcription of *sodF *gene only in conjugation with nickel-binding protein SrnQ [36]. SrnQ binds nickel but it did not show any homology to the SmtB/ArsR family, while SrnR has a DNA-binding motif but did not reveal any metal-binding capacity [36]. Our analysis indicates that Vc-HlyU does not have any metal-binding sites similar to any proteins in this family.

### Dimerization interface and inter-subunit contacts

Most SmtB/ArsR like metalloregulators form homodimers, and the dimeric interface is formed by helix 5 (α5) and the N-terminal part of the protein. The interface formed between two protein subunits provides the context for understanding the principles of molecular recognition. We analyzed the characteristics of homodimeric interfaces of SmtB, CzrA, CadC, and Vc-HlyU using the PROFACE server [37], which dissects a given protein-protein interface and obtains various parameters to characterize it. The results are shown in Table 3. The buried interface areas between the subunits are 3924 Å^2^, 3016 Å^2^, 4614 Å^2^, and 3753 Å^2 ^in SmtB, CzrA, CadC, and Vc-HlyU respectively. CadC has the largest interface area due to the presence of an additional α-helix at the N-terminus which interacts with the other monomer in the CadC dimer. The other parameters like interface area/surface area, fraction of non-polar atoms, non-polar interface area etc. of SmtB, CzrA, and CadC are found to be very similar with Vc-HlyU (Table 3). Overall the dimerization interface is highly hydrophobic in Vc-HlyU as it has a latge non-polar interface area (2526 Å^2^), similar to the other members of the family (Table 3). Vc-HlyU also has three 'self-contacting' residues L25, M95, and L98; SmtB has two F40 and L110; CzrA has three F20, T89, and M90; CadC also has three L36, I107, and I110. In a 2-fold symmetry relating the protein subunits, a residue close to the 2-fold axis may interact with the same residue from the other subunit – thus making up a pair of 'self-contacting' residues; these 'self-contacting' residues are found to be important in forming homodimeric interface [38].

Interestingly, HlyU with a long N-terminal His_6_-tag found to be monomer in solution [32], but on cleaving the His_6_-tag HlyU forms a dimer (Saha & Chakrabarti, unpublished results). As the N-terminal region is found to be important in forming dimeric interface, most probably the His_6_-tag was creating some kind of hindrance to the formation of the dimer. All these results suggest that Vc-HlyU is also a homodimer, as found in the case of other SmtB/ArsR family members.

### Protein-DNA interactions

Homology-modeled structures may be of too low resolution to characterize the protein-DNA contacts at the atomic level and elucidate their mechanism of action, but they can suggest which sequence regions or individual amino acids are essential components of the binding surfaces. In particular, identification of amino acids potentially involved in protein-DNA contacts may guide mutagenesis experiments aimed at the engineering of protein variants with novel specificities and mechanisms.

**Figure 4:**
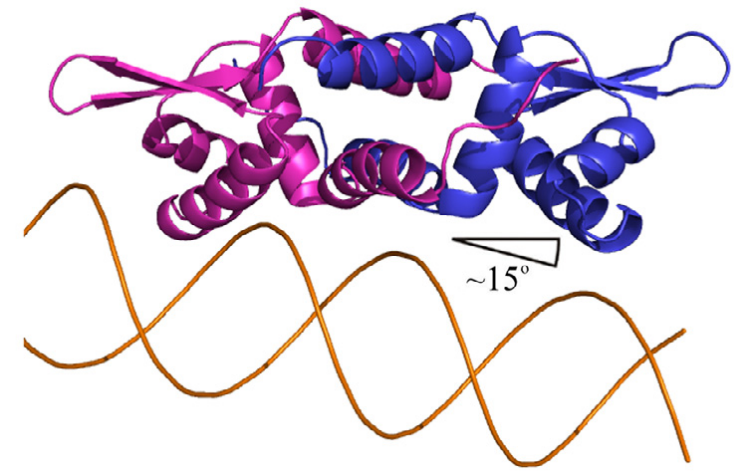
Model of Vc-HlyU binding to DNA based on the similarity of Vc-HlyU with OhrR DNA-recognition motif. Monomers A and B are in magenta and blue color respectively.

Vc-HlyU is predicted to be a winged-helix DNA binding protein. The two wings (W1 and W2), three α-helices (α2, α3, and α4), and two β-strands (β1 and β2) arranged in an order α2-α3-α4-β1-W1-β2-W2, to form a typical winged-helix motif [39,40]. The putative DNA-binding domain has a helix-turn-helix motif consisting of α3-turn-α4. When involved in DNA-binding, the recognition helix (α4) might interact with the major groove of a duplex DNA, as suggested by other winged-helix protein-DNA co-crystal structures [41]. The wing W1 is predicted to interact with the adjacent minor groove. To find out the protein-DNA interaction we created a simple model of Vc-HlyU binding to DNA, based on the similarity of the DNA-recognition motif of Vc-HlyU with the winged helix-turn-helix motif of the MarR-family transcription regulator, OhrR from *Bacillus subtilis *[42]. The coordinates of Vc-HlyU DNA-recognition motif superimposed onto those of OhrR, and the actual DNA in OhrR was replaced by an idealized B-DNA model. The model shows that the binding of both ends of the Vc-HlyU dimer would require a bending of the DNA-helix of about 15° (Figure 4). Based on the homology to OhrR the residues which may be important for DNA-binding are in α1 (K26 and A27), N30, in α2 (E31, R32, and R33), E44, S46, in α3 (V47 and G48), S57, in α4 (Q58, S59, A60, S62, Q63, A66, W67, and R70), in β1 (T76 and K78), Q81, T82, in β2 (V83), Y85. The positions of these residues which may be required for DNA recognition in Vc-HlyU are quite similar to what was predicted in case of SmtB [31]. The proposed model would allow binding of each end of the Vc-HlyU dimer to consecutive major grooves.

**Figure 5:**
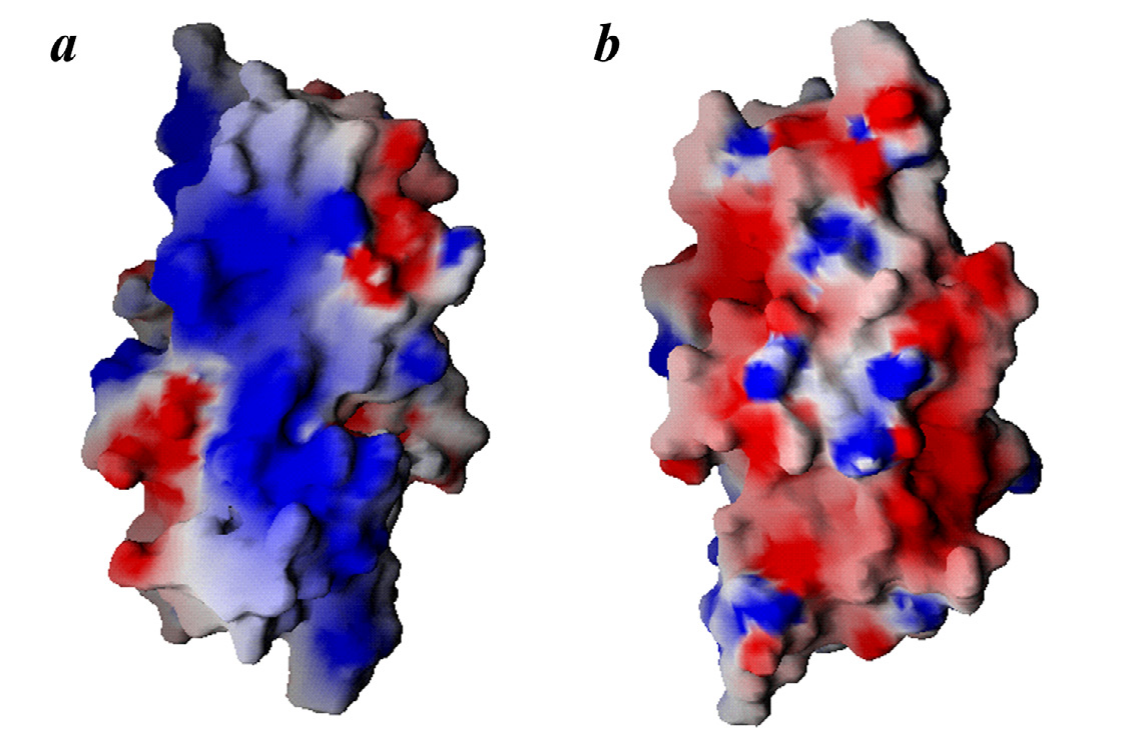
The electrostatic surface potential of the Vc-HlyU dimer with blue and red regions indicating positive and negative electrostatic regions, respectively. (a) The DNA-binding surface indicates the highly basic nature of the DNA-binding domains in the two subunits. (b) The molecule is rotated 180° around the long axis showing the opposite surface of DNA-binding region which is predominantly negatively charged and highly acidic.

Figure 5 shows the electrostatic potential of the Vc-HlyU dimer computed by GRASP [43]. A positive charge is found in regions of the surface directly involved in protein-DNA interaction (Figure 5a) and the overall charge distribution at the DNA-binding surface is quite similar to the other SmtB/ArsR family members. The HTH motif is the most positively charged region of the DNA-binding domain. This is in accordance with the postulated role of the DNA-binding domain in the interaction with the phosphate backbone of DNA. In contrast, the solvent accessible surface of Vc-HlyU is negatively charged (Figure 5b).

### Evolutionary analysis

**Figure 6:**
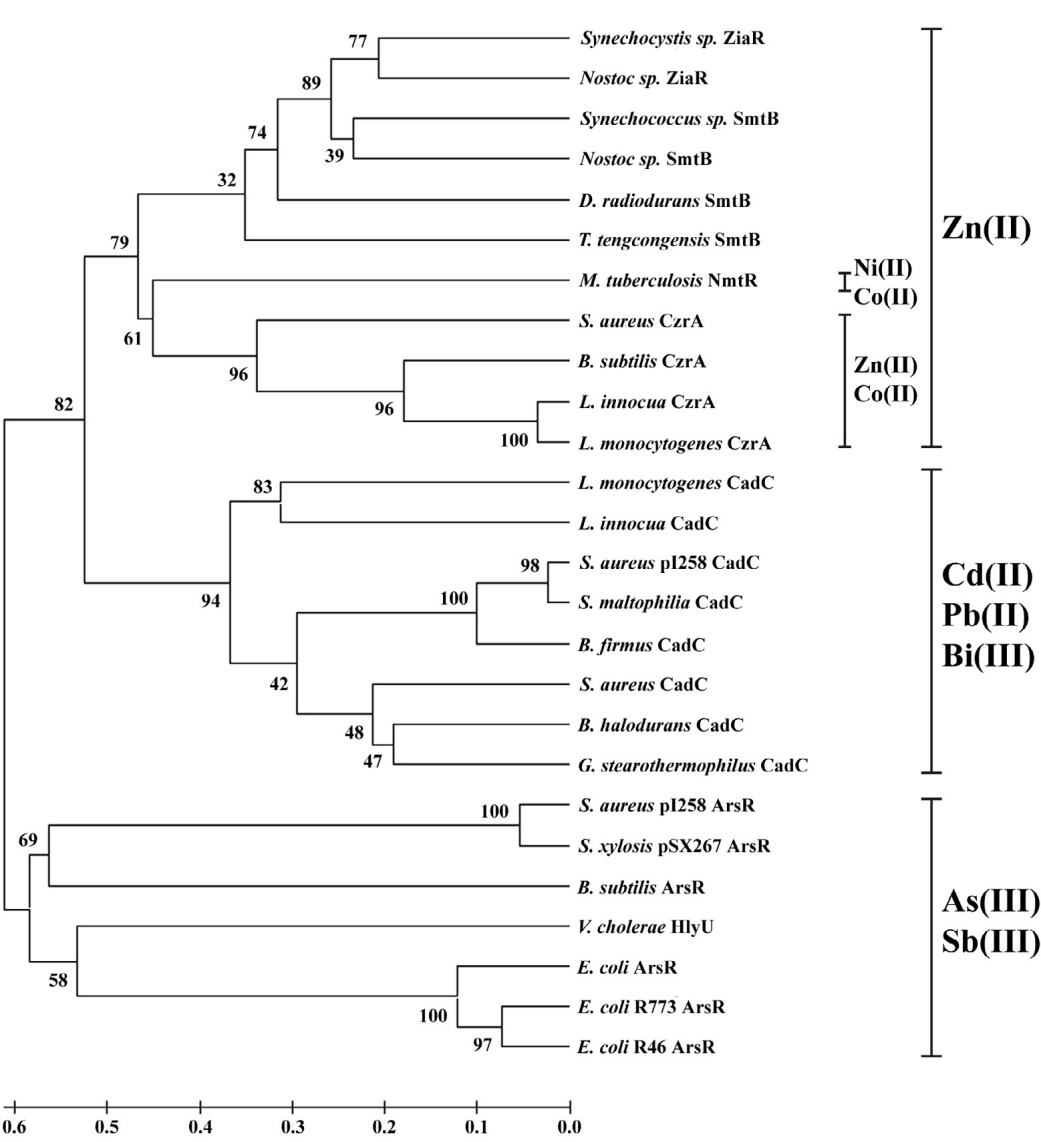
Phylogenetic relationship of SmtB/ArsR family members (25 SmtB/ArsR family members and the Vc-HlyU sequence). This is a bootstrap consensus tree based on 1000 replicates. The numbers on the nodes are bootstrap values. The metal-binding motifs sense different metals, present in the three major clusters, are indicated on the right.

A phylogenetic analysis of a subset of SmtB/ArsR repressor sequences clearly showed that the sensors that respond to the biologically required metal ions cluster on a distinct branch of the dendrogram and may have evolved later than those which confer resistance to the environmental stress resulting from the heavy metal pollutants [3]. The members most closely related to a common ancestor, represented by the ArsR, contain only the first metal binding site, while the more divergent homologue, CzrA possesses only the α5 metal binding site. CadC, with both types of metal binding sites, might represent an evolutionary intermediate between ArsR and SmtB [33]. To find out the evolutionary relationship between Vc-HlyU and SmtB/ArsR family members a phylogenetic tree of 26 sequences (25 SmtB/ArsR family member sequences, taken from reference no. 3, and the Vc-HlyU sequence) was created using the neighbor-joining, minimum evolution and UPGMA methods. The distance estimation was done using the Poisson correction method. Neighbor-joining, minimum evolution and UPGMA analyses produced topologically identical trees. Bootstrap analyses were performed on the neighbor-joining, minimum evolution and UPGMA trees with 1000 replications. All phylogeny trees were constructed using "MEGA version 3.1", a molecular evolutionary genetic analysis software [44]. The α3N and α5 sensors appear to cluster on separate nodes of the dendrogram and linked by a common evolutionary ancestor. Vc-HlyU was found to cluster along with ArsR proteins, which are considered as the evolutionary primitive and the founder members of the SmtB/ArsR family of proteins (Figure 6). The clustering of Vc-HlyU along with ArsRs suggests that Vc-HlyU is close to the common ancestor from which this family of proteins evolved. These data suggest that during evolution Vc-HlyU probably occurred by gene duplication followed by mutational events that led to the loss of the metal-binding residues.

## Conclusion

In recent years, the methodology to predict the 3D structure of a protein starting from its sequence has improved in accuracy and statistical robustness [45]. Though the conservation of the structure can be inferred from the high sequence similarity, it is well known that in a single family the function and the fold can be retained even if proteins have a low sequence similarity. Even if the sequence identity between the target and template proteins is lower than 40%, the secondary structural information can be used for sequence alignment, and the strategy of comparative modeling can be applied with success [46]. In this paper, we present results of modeling of Vc-HlyU with a comparative modeling strategy, starting from the 3D structures of proteins belonging to the same functional and structural family. The results show that *Vibrio cholerae *transcription regulator HlyU maintains similar fold as that of SmtB/ArsR family of repressor proteins, but lost the key metal binding residues.

The SmtB/ArsR family of metalloregulators responds to a wide variety of metals. The metal-dependent transcriptional regulation is the major mechanism of the cellular response to changing metal concentrations. It is, therefore, crucial to understand how metalloregulators are able to differentiate between metals and how this information is translated into transcriptional control. The ancestral member of this family, ArsR had only the first metal-binding site. During evolution, SmtB retained the site partially, but lost the function, while CzrA lost the site completely. However, both acquired a regulatory role for the metal binding at a new site (α5). CadC, which possesses both the metal binding sites, might represent an evolutionary intermediate between ArsR and SmtB. CmtR binds metal at a unique site at α4 helix, while another related protein SrnR senses metals but only in conjugation with metal-binding protein SrnQ. Using these sites the members of this family respond to an amazing array of different metals and metalloids, suggesting that during evolution one could acquire or lose one or more metal binding sites. However, there is also a possibility that during evolution one could lose all the metal binding sites and show an entirely different function, or vice versa. The structure of Vc-HlyU suggests that the evolution of Vc-HlyU probably occurred by gene duplication and mutational events that led to the loss of the metal binding sites, and eventually it acquired a function that is seemingly different from the repressors constituting the SmtB/ArsR family. HlyU is a positive regulator of *hlyA*, but there is no biochemical evidence that HlyU binds to the *hlyA *O/P region. Therefore it is still uncertain if HlyU is a transcriptional activator or the regulation observed [13,14] is due to the involvement of unknown intermediary which is repressed by HlyU. Experiments are underway to determine the DNA-recognition, if any, at the O/P sequence of *hlyA *gene. The present work exemplifies how the same fold can have different functions depending on the presence or absence of metal-binding sites.

## Methods

### Fold recognition and sequence alignments

Sequence searches of the non-redundant (nr) database were carried out at NCBI using PSI-BLAST [47], using the *Vibrio cholerae *HlyU (Vc-HlyU) sequence as a query. Secondary structure prediction and tertiary fold-recognition were carried out using the GeneSilico meta-server gateway [48]. The secondary structure was predicted using SAM [49], PSIPRED [50], JNET [51], SABLE [52], PROF [53], JUFO [54], and PROFsec [55]. The fold-recognition analysis was carried out using FFAS03 [56], INBGU [57], mGENETHREADER [58], SPARKS [59], FUGUE [60], and 3DPSSM [61]. The fold-recognition alignment reported by these methods were compared, evaluated, and ranked by Pcons server [62]. The analysis of architectural motifs and the topology of proteins with known three-dimensional structure was made according to SCOP [63] and CATH [64] classifications. PROSITE [65] database was used for searching functional motifs. Multiple alignments were generated using CLUSTALW [66] and subsequently subjected to minor manual editing.

### Homology modeling and data analysis

After careful examination of potential templates the structure of SmtB from *Synechococcus *PCC7942 (PDB entry: 1smt) [31], CzrA from *Staphylococcus aureus *(PDB entry: 1r1u) [67], and CadC from *Staphylococcus aureus *pI258 (PDB entry: 1u2w) [33] were selected for homology modeling. A pairwise alignment between Vc-HlyU and the template sequences were manually adjusted taking into consideration multiple sequence alignments, structural alignments and the continuity of secondary structure elements. A few manual refinements were added to account for the position of secondary structures. For the modeling procedure, only the region in the sequence for which the 3D structure of the template is available was considered. As a consequence, we excluded from the model of Vc-HlyU the first 8 amino acids as well as the last residue due to the unavailability of template structure. The alignments between the sequence of Vc-HlyU and the structures of the selected templates were used as a starting point for modeling of the HlyU tertiary structure comprising cycles of model building by MODELLER v8.1 [68]. The best models among those obtained were chosen by evaluating the stereochemical quality with the program PROCHECK [23], and side-chains were optimized using SCWRL 3.0 [69]. Secondary structures on the final 3D model were calculated with the program DSSP [70], and solvent accessibility of the amino acids was calculated with the program NACCESS [71].

## Availability

Atomic coordinates for *Vibrio cholerae *transcriptional regulator HlyU are publicly available via the PMDB database [72] as a theoretical model (PMDB id: PM0074675).

## Authors' contributions

RPS carried out the fold-recognition analysis, built the models, and identified the functionally important residues. RPS and PC participated in interpretation of the data and writing the manuscript. Both the authors have read and accepted the final version of the manuscript.

## Supplementary Material

Additional file 1 Prosa energy plot of the Vc-HlyU model along with template structures. Click here for file

## Figures and Tables

**Table 1:** Proteins found with BLAST search producing alignments (*E *value ≤ 1 × 10^-20^) with the sequence of HlyU from *V. cholerae *are shown together with proteins with known 3D structure

Genebank code	Protein description	Organism	Score (bits)	*E*	Sequence identity (%)
ZP_00992718.1	Transcription activator HlyU	*Vibrio splendidus*	137	2 × 10^-31^	82
ZP_01065735.1	Transcription activator HlyU	*Vibrio *sp.	135	4 × 10^-31^	82
NP_759503.1	Transcription activator HlyU	*Vibrio vulnificus*	132	6 × 10^-30^	82
NP_796908.1	Transcription activator HlyU	*Vibrio parahaemolyticus*	130	2 × 10^-29^	83
ZP_01261951.1	Transcription activator HlyU	*Vibrio alginolyticus*	129	5 × 10^-29^	83
ZP_00761653.1	Predicted transcriptional regulator	*Vibrio *sp.	129	6 × 10^-29^	83
YP_128811.1	Putative transcription activator HlyU	*Photobacterium profundum*	126	3 × 10^-28^	75
YP_203846.1	Transcription activator HlyU	*Vibrio fischeri*	124	2 × 10^-27^	73
NP_719085.1	Transcription activator HlyU	*Shewanella oneidensis*	109	4 × 10^-23^	64
YP_751572.1	Transcriptional regulator, ArsR family protein	*Shewanella frigidimarina*	107	1 × 10^-22^	63
ZP_01437145.1	Transcriptional regulator, ArsR family protein	*Shewanella baltica*	106	2 × 10^-22^	63
ZP_00838999.1	Regulatory protein, ArsR	*Shewanella *sp.	105	5 × 10^-22^	63
YP_563729.1	Regulatory protein, ArsR	*Shewanella denitrificans*	105	5 × 10^-22^	63
ZP_01305695.1	Transcription activator HlyU	*Oceanobacter *sp.	101	1 × 10^-20^	58
Proteins with known 3D structure^a^					
PDB code: 1u2w	Cadmium efflux system accessory protein CadC	*Staphylococcus aureus*	46.2	2 × 10^-06^	34
PDB code: 1r1u	Repressor protein CzrA^b^	*Staphylococcus aureus*	45.4	3 × 10^-06^	33
PDB code: 1smt	Transcriptional repressor SmtB^b^	*Synechococcus *sp.	36.2	0.002	37

^a ^Found from a BLAST search on PDB.

^b ^A few other closely related structures have PDB codes 1r1v and (1r22, 1r23 and 1r1t).

**Table 2:** Evaluation of the quality of the model using some statistically derived methods

				Solvation Free Energy (*SFE*)	
				
Protein	Eval23D	Verify3D	ProsaII	Calculated^a^	Predicted^b^
Vc-HlyU	4.156	0.401	-1.836	-200.9	-208.4
SmtB	4.768	0.418	-1.353	-221.1	-209.6
CzrA	5.851	0.435	-2.075	-215.9	-196.0
CadC	5.878	0.402	-1.831	-227.8	-228.8

^a ^Using Eval123D server [24].

^b ^Using the equation, *SFE *= 15.30 - 1.13 *N*, where *N *= number of residues [28].

**Table 3:** Characteristics of homodimeric interfaces

	Vc-HlyU	SmtB	CzrA	CadC
Interface area (Å^2^)	3753	3924	3016	4614
Interface area/Surface area	0.27	0.27	0.23	0.3
Number of atoms	378	392	305	455
Number of residues	95	99	73	118
Fraction of non-polar atoms	0.65	0.68	0.68	0.66
Non-polar interface area (Å^2^)	2526	3020	2221	3307
Fraction of fully buried atoms	0.35	0.44	0.43	0.44
Self-contacting residues	Leu25, Met95, Leu98	Phe40, Leu110	Phe20, Thr89, Met90	Leu36, Ile107, Ile110

Both the subunits have been indicated in the calculation.
