# Supplementary material for: Molecular modeling and characterization of Vibrio cholerae transcription regulator HlyU
Source: BMC Struct Biol. 2006 Nov 20;6:24. doi: 10.1186/1472-6807-6-24 (PMC1665450; doi:10.1186/1472-6807-6-24)
Supplement: Additional file 1 — Prosa energy plot of the Vc-HlyU model along with template structures. [file 1472-6807-6-24-S1.pdf]

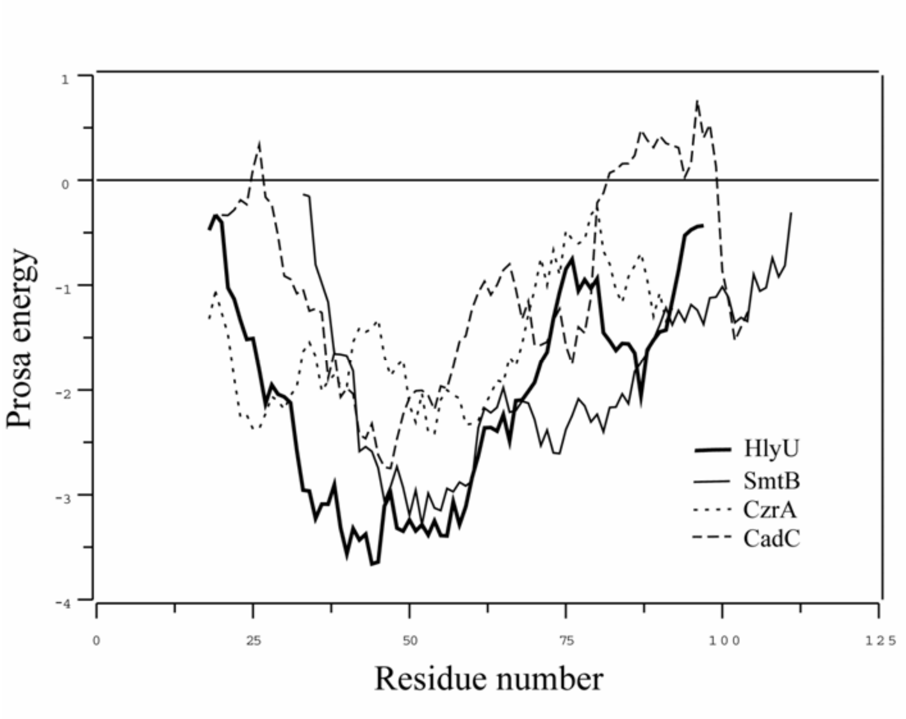

Additional file 1. Prosa energy plot of the Vc-HlyU model along with template structures. The graphs are smoothed over a window size of 20 residues. The curves represent the residue interaction energies; negative values correspond to stable parts of the molecules.
